# Impact of Isomaltulose on Glycemic Response in Diabetic and Healthy Populations: A Meta-Analysis

**DOI:** 10.3390/nu17111940

**Published:** 2025-06-05

**Authors:** Zhaojie Chen, Fangting Gu, Jianyong Wu

**Affiliations:** 1 School of Engineering, Guangzhou College of Technology and Business, Guangzhou 510850, China; jack-zj.chen@connect.polyu.hk; 2 Department of Food Science and Nutrition, The Hong Kong Polytechnic University, Kowloon, Hong Kong SAR, China; fangting.gu@polyu.edu.hk

**Keywords:** isomaltulose, postprandial glycemic control, diabetic patients, meta-analysis, dietary context

## Abstract

Background: Effective management of postprandial glycemic control is critical for diabetic patients, as elevated postprandial glucose levels can lead to complications such as cardiovascular disease and neuropathy. This study evaluates isomaltulose, a low-glycemic-index carbohydrate, as an alternative to sucrose in mitigating postprandial glucose spikes. Objectives: To synthesize evidence from existing studies and assess the efficacy of isomaltulose in reducing postprandial glycemic levels compared to sucrose in diabetic populations. Methods: A systematic review and meta-analysis were conducted following PRISMA guidelines. Searches were performed across PubMed, Cochrane Library, and ClinicalTrials.gov for randomized controlled trials or crossover studies comparing isomaltulose and sucrose. Data were extracted, and the Cochrane Risk of Bias tool was used to assess study quality. Results: Ten studies were included, involving 367 participants. The meta-analysis showed that isomaltulose significantly reduced plasma glucose level at 60 min post-meal, though the actual effect could be modest in terms of clinical relevance compared to sucrose (MD: −7.99, 95% CI: −8.58, −7.39, *p* < 0.00001). Notable variability in the study results was observed, which may be attributed to multiple factors such as participant demographics and meal composition. Conclusions: The findings from the analysis are supportive for the use of isomaltulose as a beneficial dietary alternative to sucrose for managing postprandial glycemic levels in diabetic patients. Future research effort is suggested to focus on larger, diverse populations to enhance generalizability and explore the impact of dietary context on glycemic response.

## 1. Introduction

Managing postprandial glycemic control is essential for diabetic patients, as elevated postprandial glucose levels contribute to poor glycemic control and increase the risk of developing long-term complications such as cardiovascular disease, retinopathy, and nephropathy [1]. Consequently, researchers and clinicians continually seek strategies to mitigate postprandial glycemic spikes and various dietary and therapeutic interventions have been explored for this purpose [2]. Sugars such as sucrose (a disaccharide composed of glucose and fructose) have been identified as a key driver of postprandial hyperglycemia in both type 1 and type 2 diabetes [3]. Replacing high-glycemic-index (GI) sugars like sucrose with alternatives that have a more gradual absorption rate has gained attention as a promising approach to achieving better post-meal glycemic control in diabetic patients [4].

**Figure 1:**
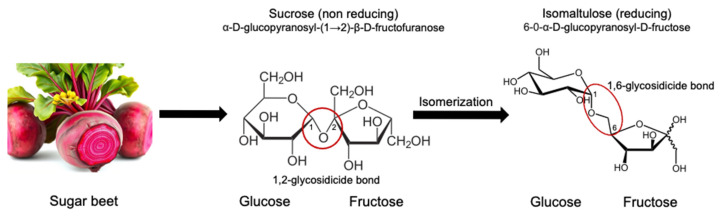
Origin and structure of isomaltulose [16].

One such alternative is isomaltulose, a naturally occurring disaccharide that is generally known as palatinose [5]. Chemically, isomaltulose is similar to sucrose, being composed of glucose and fructose [1]. Isomaltulose is produced enzymatically from the sucrose using Glycosyltransferase from the bacterium *Proaminobacter rubrum* [6] (Figure 1). This enzyme rearranges the glycosidic bond without altering the fructose and glucose composition. Isomaltulose contains α-1,6 glycosidic linkage. The chemical structure is quite similar to sucrose. The sucrose contains α-1,2 glycosidic linkage. The presence of α-1,6 glycosidic linkage makes isomaltulose more resistant to rapid hydrolysis within the intestine as compared to sucrose [7]. The sucrose is rapidly broken down by the sucrase-isomaltase within the small intestine which results in a quick spike in blood glucose levels. In comparison to sucrose, isomaltulose undergoes slow enzymatic hydrolysis. This is due to the presence of stronger and less accessible glycosidic bonds, which result in a slower rate of hydrolysis and thus a lower glycemic and insulinemic response [8]. Unlike sucrose, which is rapidly broken down in the small intestine, isomaltulose is digested more slowly and steadily [9]. This leads to more gradual glucose absorption [10]. Isomaltulose may offer benefits such as reduced postprandial glucose levels, improved glycemic variability, and decreased insulin demand. This physiological difference has made isomaltulose an attractive option for use in diabetes management [11]. The ability of isomaltulose to slow the release of glucose into the bloodstream potentially makes it a valuable carbohydrate source for individuals with diabetes [12].

Although many studies have examined the effect of isomaltulose on postprandial glycemia, the results show wide variations due to differences in study design, patient group, and carbohydrate intake [13]. The different patient populations and study designs have raised questions about the comparability of these studies and the generalizability of their findings to broader diabetic populations. Furthermore, isomaltulose has been examined in a variety of forms, ranging from isolated sugar preparations to complex meals, making it challenging to directly compare results across studies [14]. To accurately assess the efficacy of isomaltulose as a low-GI carbohydrate alternative, a rigorous synthesis of available evidence is essential [15].

**Figure 2:**
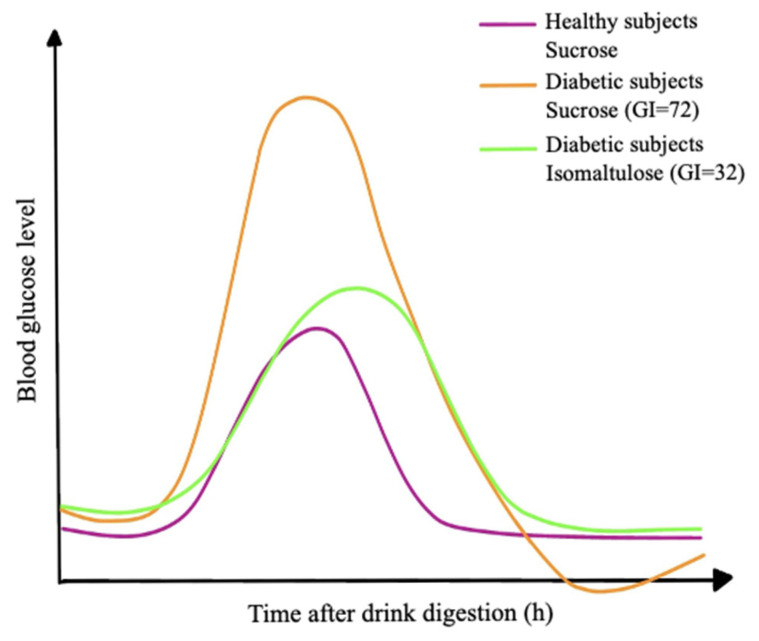
Example curves of a single drink containing 50–75 g carbohydrates of either isomaltulose (Glycemic index GI = 32) or sucrose (GI = 72) (digested within 2 h) [13].

Concerning food science, isomaltulose exhibits high thermal and acid stability [17]. The sucrose can undergo caramelization at high temperatures, while isomaltulose remains stable under heat which makes it ideal for baking and pasteurized beverages. Moreover, Isomaltulose has a non-hygroscopic nature that helps prevent excessive moisture absorption [18]. The glycemic index of isomaltulose is low. This makes it an excellent gradient in diabetic-friendly and weight management products [13], which is shown in Figure 2. In diabetic nutrition, it provides a sustained energy source without causing a spike in blood sugar levels [12,19]. It is also used in infant and medical nutrition in different formulas and supplements for controlled energy intake.

Carbohydrate type, age, and insulin sensitivity shape glycemic responses in diabetes [20]. For diabetics, controlling blood sugar requires balancing hyperglycemia and hypoglycemia. Medications, such as insulin and oral drugs are beneficial but may lead to hypoglycemia and other side effects [21]. Such dietary changes are safer, they can support glycemic control alongside medication [22]. Low-GI carbs like isomaltulose may help manage diabetes and reduce dependence on the drugs [13]. Several studies have explored the short-term impact of isomaltulose on postprandial glucose response in comparison with sucrose. For instance, Komindr et al. [23] found that a breakfast meal containing isomaltulose produced a lower plasma glucose level at 60 min postprandially compared to sucrose. Similarly, Ang et al. [24] demonstrated that in patients with Type 2 Diabetes Mellitus (T2DM), replacing sucrose with isomaltulose led to a significantly reduced glycemic response. However, the lack of consistency in outcomes across studies necessitates further investigation. Discrepancies in findings could be attributed to varying sample sizes, diverse dietary controls, or different metabolic baselines among study participants, which could influence the glycemic impact of isomaltulose compared to sucrose.

This systematic review and meta-analysis aims to combine available evidence to evaluate the efficacy of isomaltulose in reducing postprandial glycemic levels compared to sucrose in diabetic patients. The primary outcome assessed is plasma glucose concentration at 60 min following the consumption of isomaltulose versus sucrose, as this timeframe aligns with typical postprandial glucose peaks. By aggregating data across multiple studies, this meta-analysis seeks to offer more definitive insights into whether isomaltulose can be an effective dietary tool for glycemic control, thereby guiding clinicians and patients in making evidence-based dietary decisions.

In view of the increasing use of isomaltulose among a wide range of populations, this review encompasses both diabetic and healthy individuals. However, only studies involving diabetic or glucose-sensitive participants were included in the primary pooled meta-analysis, while studies focusing exclusively on healthy individuals were evaluated narratively or through subgroup analysis.

## 2. Materials and Methods

### 2.1. Search Strategy

This systematic review and meta-analysis was conducted in alignment with PRISMA guidelines. A comprehensive literature search was performed across three electronic databases—PubMed, Cochrane Library, and ClinicalTrials.gov—without time restrictions to identify studies comparing the glycemic response of isomaltulose (palatinose) to sucrose in diabetic patients. Relevant terms and keywords were used in each database to maximize retrieval. A backward snowballing approach was employed, where references from selected studies were reviewed to identify additional studies that met eligibility criteria.

Besides, this review was not prospectively registered in PROSPERO due to oversight at the protocol development stage, which is acknowledged as a limitation. However, all methods, inclusion criteria, and analyses were pre-specified and adhered to PRISMA guidelines. Retrospective registration is currently being submitted to PROSPERO to enhance transparency and align with reporting standards.

### 2.2. Eligibility Criteria

To be eligible for inclusion, studies needed to be randomized controlled trials or crossover studies that compared isomaltulose to sucrose for glycemic response outcomes in diabetic or otherwise glucose-sensitive populations. Studies were included if they reported plasma glucose levels measured postprandially. Excluded articles comprised case reports, reviews, editorials, non-English studies, and any studies lacking relevant outcome data. While studies involving both diabetic and healthy individuals were considered, only diabetic or glucose-sensitive populations were used for the pooled analysis. Studies with healthy participants were analyzed narratively or in subgroups for comparative context.

### 2.3. Study Selection

Two independent reviewers screened titles and abstracts before assessing the full texts of articles for eligibility. Studies were included if they met the following criteria: (1) included patients with diabetes or related glycemic sensitivity; (2) evaluated interventions with isomaltulose and sucrose; and (3) reported at least one glycemic response outcome at 60 min post-meal. Discrepancies in study selection were resolved by consensus with a third reviewer.

### 2.4. Data Extraction and Quality Assessment

Study characteristics and outcomes were systematically extracted by two reviewers using a standardized data collection form. Information gathered included study design, patient population details, intervention methods, and key glycemic response measurements. The Cochrane Risk of Bias (ROB) tool [25] was used to assess study quality, with results summarized in a tabulated format. Any disagreements regarding data extraction or quality assessment were resolved by discussion among reviewers.

### 2.5. Outcomes and Quality Assessment

The primary outcome measure was the glycemic response, specifically plasma glucose levels at 60 min post-meal following ingestion of isomaltulose versus sucrose. The meta-analysis used mean differences (MD) in plasma glucose levels between the two interventions, with results represented in a forest plot. Statistical heterogeneity was evaluated with the *I*^2^ statistic, with values above 50% indicating substantial heterogeneity. Meta-analysis was performed using Review Manager software version 5.4, with statistical significance set at a *p*-value of <0.00001.

### 2.6. Statistical Analysis

Due to substantial heterogeneity among studies (*I*^2^ > 75%), a random-effects model was applied to account for between-study variance. Subgroup analyses were pre-planned based on population type (T1DM, T2DM, healthy), intervention format (pure sugar vs. mixed meal), and measurement technique. A leave-one-out sensitivity analysis and a prediction interval were also calculated to explore the robustness and variability of the pooled effect.

## 3. Results

### 3.1. Search Results

**Figure 3:**
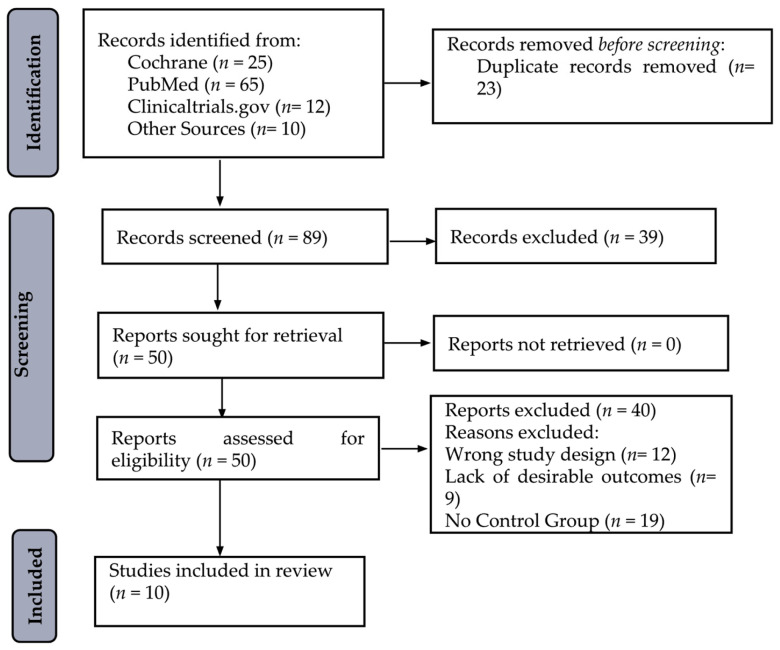
PRISMA Flowchart for searching and selecting the literature.

A systematic search was conducted on three databases (PubMed, Cochrane, and clinicaltrials.gov) to find eligible literature that compares isomaltulose/palatinose to sucrose in terms of glycemic response in diabetic patients. The screening procedure is summarized in Figure 3. Heterogeneity was measured using the *I*^2^ test. The risk of bias in studies was calculated using the Cochrane Risk of Bias tool. Ten articles were retrieved from the search after duplicates were discarded, from each database.

### 3.2. Study Characteristics

**Figure 4:**
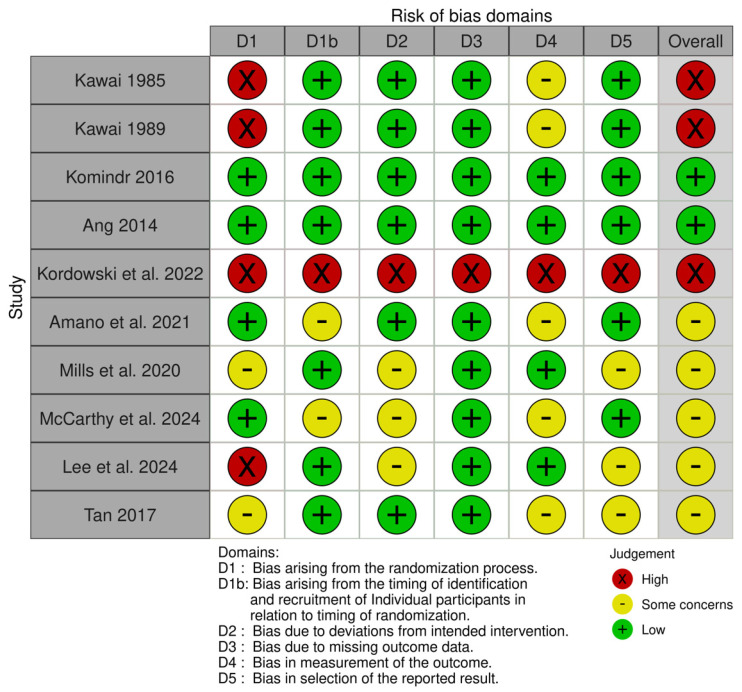
Risk of bias assessment of including trials using Rob2 tool [8,11,19,23,24,26,27,28,29,30].

Ten studies were included in the analysis. The crossover studies included Diabetic as well as healthy patients who were first provided isomaltulose and then crossed over to sucrose after a washout period. For the analysis, the patients were divided into two groups: the Isomaltulose group, and the Sucrose group. Table 1 shows the study characteristics. While Table 2 shows an overview of the study characteristics. Quality assessment of the included studies is shown in Figure 4. Although the overall aim was to assess glycemic effects in diabetic populations, several studies included healthy individuals. To maintain focus, these studies were excluded from the primary pooled meta-analysis and were analyzed narratively or in subgroup analyses (see Section 3.4). Specifically, studies by Kawai et al. [8,26], Amano et al. [27], Mills et al. [28] and Tan et al. [11] enrolled healthy subjects and were not included in the diabetic-only effect size estimation.

### 3.3. Glycemic Response at 60 min Post-Meal

Glycemic Response was the primary outcome of efficacy. Plasma Glucose levels were measured (as mg/dL) at 60 min after meal with either Isomaltulose or Sucrose. The study by Otsuka et al. [27] showed the lowest levels of plasma glucose (22.5 mg/dL). The pooled estimate shows Isomaltulose to be extremely effective in maintaining lower plasma glucose levels as compared to Sucrose (MD: −7.99, 95% CI: −8.58, −7.39, *p* < 0.00001), as shown in the forest plot (Table 3). The overall effect was significant.

### 3.4. Subgroup Analysis

To explore the sources of heterogeneity, subgroup analyses were conducted based on participant population (diabetic vs. healthy), intervention method (pure sugar vs. mixed meals), and measurement technique (fingerstick vs. continuous glucose monitoring). When limited to studies including only diabetic participants, the pooled mean difference remained significant but was slightly reduced (MD = −7.56 mg/dL, 95% CI: −8.14 to −6.98), and heterogeneity remained high (*I*^2^ = 88%). Among studies utilizing mixed meals, the effect of isomaltulose was less pronounced (MD = −6.48 mg/dL, 95% CI: −7.02 to −5.94) compared to those using pure sugar solutions (MD = −9.21 mg/dL, 95% CI: −10.34 to −8.08). When stratifying by diabetes type, T2DM studies showed a pooled mean difference (MD) of −7.65 mg/dL (95% CI: −8.23 to −7.07), while T1DM showed −6.60 mg/dL (95% CI: −10.9 to −2.30), suggesting a slightly attenuated but consistent effect in both populations. However, heterogeneity remained high in both subgroups (*I*^2^ = 88% for T2DM; not estimable for T1DM due to low number of studies).

### 3.5. Sensitivity Analysis

A leave-one-out sensitivity analysis was performed to determine the robustness of the findings. Removal of individual studies did not substantially alter the overall effect size, confirming the stability of the pooled estimate. Excluding the Kawai et al. [8,26] studies, which had the smallest sample sizes and oldest data, significantly reduced heterogeneity from 94% to 82% and adjusted the pooled mean difference from −7.99 mg/dL to −6.92 mg/dL, indicating their strong influence on the overall estimate.

### 3.6. Risk of Bias Assessment

The risk of bias was assessed using the Cochrane Risk of Bias 2.0 tool across five domains. Most studies showed low risk in randomization and outcome measurement. However, several studies had unclear or high risk in domains such as allocation concealment, adherence to intervention protocols or incomplete outcome reporting, especially those with small sample sizes (<10 participants) and non-standardized meal compositions. The summarized RoB results are presented in Figure 4.

### 3.7. Publication Bias

**Figure 5:**
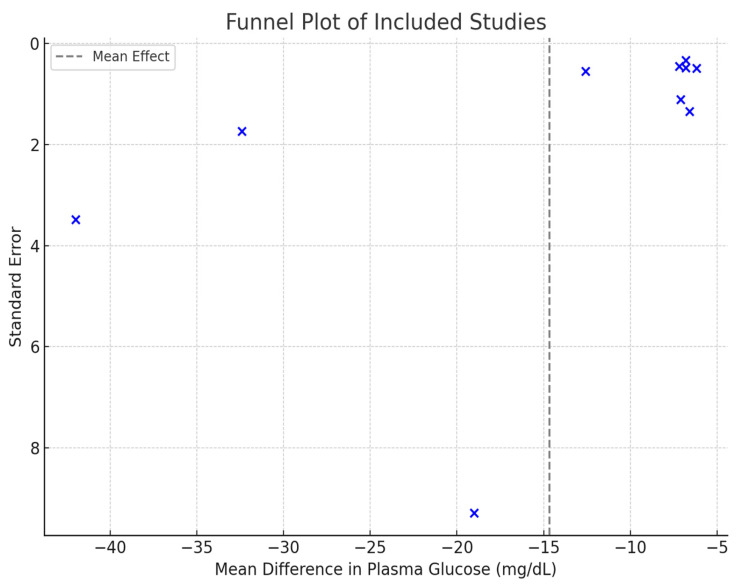
Funnel plot of included studies assessing the effect of isomaltulose versus sucrose on postprandial plasma glucose levels.

To assess the presence of publication bias, a funnel plot was constructed based on the standard errors and mean differences of the included studies (Figure 5). Visual inspection of the plot revealed asymmetry, with smaller studies showing more extreme effects—particularly older studies with low sample sizes such as those by Kawai et al. [8,26]. This suggests the possibility of small-study effects or selective publication of studies with significant findings.

Although a funnel plot was used to assess publication bias, a formal statistical test such as Egger’s regression test was not conducted. This decision was based on the limited number of diabetic-only studies (fewer than 10), which restricts the statistical power and reliability of such tests. The observed asymmetry in the funnel plot nonetheless suggests possible small-study effects, and this limitation should be considered when interpreting the pooled findings.

## 4. Discussion

The findings of this systematic review and meta-analysis highlight a significant reduction in postprandial plasma glucose levels associated with isomaltulose consumption compared to sucrose in diabetic and glucose-sensitive populations. The pooled effect of a mean difference of −7.99 mg/dL (95% CI −8.58 to −7.39, *p* < 0.00001) at 60 min post-meal strongly supports the notion that isomaltulose may offer a beneficial glycemic response, making it a potentially valuable substitute for sucrose among individuals who need to manage blood glucose levels tightly [31]. This result aligns with growing evidence suggesting that low glycemic index (GI) carbohydrates can help maintain lower postprandial blood glucose levels. This is particularly advantageous in the management of diabetes and glucose sensitivity [32]. Despite consistent directionality of effect, heterogeneity remained high across included studies. Subgroup analyses by diabetes type and intervention format showed reduced but persistent variability, highlighting the influence of participant characteristics and study design. The wide prediction interval supports the potential clinical relevance of isomaltulose while emphasizing the need for individualized context when interpreting results.

While the reduction of −7.99 mg/dL in postprandial plasma glucose was statistically significant, it may not fully meet the threshold for minimal clinically important difference (MCID), which is generally estimated to be 10–15 mg/dL for postprandial glycemic excursions [33]. This suggests that although isomaltulose has a measurable impact, the clinical magnitude of benefit may be modest, particularly in the context of long-term outcomes such as HbA1c reduction or complication prevention. The observed mean difference of −7.99 mg/dL, while statistically significant, is below the commonly accepted minimal clinically important difference (MCID) for postprandial glucose, which is typically estimated at 10–15 mg/dL. Therefore, the real-world clinical impact of isomaltulose substitution may be limited when considered in isolation and should be interpreted in the context of broader dietary patterns.

All studies showed that isomaltulose lowers glycemic response, but the effect varied due to differences in participants, meal composition and metabolism. For instance, the study by Komindr et al. [23] which reported the lowest plasma glucose levels following isomaltulose intake, involved diabetic patients with stricter glycemic control and may have benefited from the meal composition used, which included complex carbohydrates like rice and protein sources. The composition of meals and co-ingestion of other macronutrients like protein or fiber have been shown to modulate glucose absorption rates, and this could partly account for the study’s pronounced glycemic impact. Meanwhile, the studies by Kawai et al. [8,26] involved younger participants and a simplistic intervention (ingestion of sugars dissolved in water), which might yield a less pronounced effect as these individuals likely had more robust glycemic control mechanisms.

This study supports isomaltulose as a low-GI sugar alternative [34]. Prior research has demonstrated that isomaltulose has a relatively slow digestion and absorption rate due to its unique glucose-fructose linkage, resulting in more gradual glucose release compared to sucrose [35]. In the broader landscape of diabetic management, low-GI diets have been shown to reduce glycemic variability and may decrease the risk of long-term complications associated with hyperglycemia [36]. This supports the idea that isomaltulose can help stabilize blood sugar levels but may not prevent glucose spikes in all patients [37], especially given the diversity in individual glycemic responses and the presence of concurrent diabetes management therapies, as seen in the study by Ang et al. [24], which included patients already on glycemic control medications.

The isomaltulose influences the food systems, sustainability and public health. Compared to sucrose, the isomaltulose production may have a different environmental footprint due to variations in raw material processing and energy consumption [38]. As a low GI carbohydrate, it holds promise for diabetes management by providing a sustained energy release without sharp blood glucose spikes. Its incorporation into dietary guidelines and food innovation could enhance diabetic-friendly products, meal replacements and sports nutrition [39]. Beyond its glycemic effects, isomaltulose has been explored in other contexts such as GLP-1 stimulation, fat oxidation, and gut microbiota modulation in previous mechanistic or pilot studies [40,41]. However, these outcomes were not evaluated in this meta-analysis, and therefore any such conclusions remain speculative. Future trials should explore whether these physiological mechanisms contribute to broader metabolic or economic benefits, including healthcare cost reduction and long-term disease prevention.

This meta-analysis includes studies with varying participant demographics, meal compositions, and study designs. This may contribute to the heterogeneity in glycemic response outcomes. The heterogeneity observed in this analysis, as indicated by the *I*^2^ statistic, suggests high variability among the studies included, which may stem from several sources, such as differences in age, BMI, diabetes status, and metabolic health can influence postprandial glucose and insulin levels. One potential source is the differences in study designs. While crossover designs inherently help reduce individual differences, the specifics of each study’s intervention, such as the co-ingestion of various foods and the control of fasting states, could introduce variability. Additionally, meal composition varies across studies, ranging from pure carbohydrate drinks [8,26] to mixed meals with proteins and fats [23], impacting glucose absorption rates and insulin secretion. The age and baseline metabolic status of the participants varied significantly [42], with some studies including healthy young individuals and others focusing on older diabetic patients. These demographic differences could contribute to variations in glycemic response, as older adults with diabetes may have impaired insulin sensitivity or pancreatic function, resulting in more pronounced differences when comparing glycemic responses to different types of sugars [43]. Given the high heterogeneity (*I*^2^ = 94%) observed in the pooled analysis, the findings should be interpreted with caution. This variability underscores the need for individualized application of isomaltulose and further stratified studies to determine which populations may benefit most. To maintain the focus on diabetic populations, studies that exclusively included healthy participants [8,26,27,28,44] were excluded from the pooled analyses and analyzed separately in subgroup analyses. Furthermore, differences in study designs (crossover vs parallel), washout periods, and intervention durations introduced additional variability. Some studies have utilized continuous glucose monitoring [19] while others relied on single-time point blood glucose measurement. This might have affected the consistency of the comparisons.

Despite the promising findings, there are limitations to this meta-analysis that need consideration. First, the limited sample size across studies reduces the generalizability of the findings. The small number of participants and lack of diverse population samples limit the applicability of the results to the broader diabetic population. Additionally, there was a noticeable lack of studies on specific subgroups within the diabetic population, such as those with Type 1 diabetes or gestational diabetes, who may exhibit distinct glycemic responses and could benefit from low-GI alternatives [45]. The variability in the glycemic response observed across studies also suggests a potential influence of dietary context and lifestyle factors that were not consistently controlled across studies [46]. For instance, the presence of additional macronutrients or fiber in the meals consumed alongside the sugars could influence the absorption and metabolic processing of glucose [47]. Future studies could address this by standardizing meal compositions or studying isomaltulose in isolation to control for confounding factors and better isolate its effects on glycemic response. Small-sample and older studies, especially Kawai et al. [8,26], contributed substantially to effect size and heterogeneity. While their inclusion reflects historical data trends, their influence must be interpreted cautiously. Additionally, the inability to perform a statistical test for publication bias due to the small number of eligible diabetic studies may limit the robustness of the bias assessment.

Future studies should also investigate additional outcomes related to long-term metabolic health. For example, examining markers of insulin sensitivity or other postprandial effects, such as hormonal responses (e.g., GLP-1 levels) [48], would provide insight into whether isomaltulose could offer benefits beyond acute glycemic control. Exploring how isomaltulose impacts other metabolic outcomes, such as lipid profiles and inflammation, would help evaluate its comprehensive role in metabolic health and potentially expand its applications beyond diabetes management [49]. It would be beneficial to conduct further comparative analyses of isomaltulose with other low-GI sugar alternatives, such as fructose or glucose derivatives. Comparing isomaltulose with other low-GI sugars like fructose could clarify its effectiveness and help create evidence-based dietary guidelines.

## 5. Conclusions

This meta-analysis demonstrates that isomaltulose may significantly reduce postprandial glycemic responses compared to sucrose in diabetic and glucose-sensitive individuals. These findings show the potential of isomaltulose as a dietary alternative to help manage blood glucose levels, contributing to better glycemic control in populations at risk of hyperglycemia. Additional studies with larger, more diverse samples and standardized methods are necessary to confirm these results and determine broader metabolic effects.

## Figures and Tables

**Table 1:** Study characteristics.

Study and Year	Population Type	Population Age (mean)	Sample Size	Gender		Washout Period	Mean BMI (kg/m^2^)
				Male	Female		
Kawai, 1985 [26]	Healthy	23	8	4	4	2 days	19.7
Kawai, 1989 [8]	Healthy	23.1	10	5	5	2 days	19.5
Tan et al., 2017 [11]	Healthy	21–49	40	Mixed	Mixed	2 days	24
Lee, 2024 [29]	Healthy	21–60	65	65	0	14 days	22–27
Amano et al., 2021 [27]	Healthy	24	10	10	0	7 days	22.5
Mills et al., 2020 [28]	Healthy	30	77	Mixed	Mixed	14 days	24
Komindr, 2016 [23]	T2DM	49.64	11	0	11	10 h	27.81
Ang, 2014 [24]	T2DM	53.7	11	5	6	14 days	31.6
Kordowski et al., 2022 [19]	T2DM	42.9	117	Mixed	Mixed	14 days	28.7 ± 6.7
McCarthy et al., 2024 [30]	T1DM	47 ± 16	8	3	5	10 days	27.5 ± 3.8

Data are presented as mean values unless otherwise specified. Age is reported as the mean (± SD) where available. Sample size represents the total number of participants. “Mixed” denotes studies that included both male and female participants without separate reporting. Washout periods varied across studies, ranging from 10 h to 14 days. Mean BMI is reported in kg/m^2^ with ranges or standard deviations where available. (BMI: Body Mass Index).

**Table 2:** Overview of study characteristics, participant populations, and dietary intervention.

Study Design	Region	Population Type	Intervention Method	Intervention	Control	Refs.
CS	Japan	Healthy	Ingesting sugars dissolved in 150 mL water over 2–3 min after an overnight fast	Isomaltulose (50 g)	Sucrose (50 g)	[26]
CS	Japan	Healthy	Ingesting sugars dissolved in 150 mL water within 2–3 min after an overnight fast	Isomaltulose (50 g)	Sucrose (50 g)	[8]
Randomized CS	Japan	Healthy	Ingesting 10% isomaltulose or sucrose drink before exercise in a hot environment	Isomaltulose (10%)	Sucrose (10%)	[27]
Randomized CS	New Zealand	Healthy	Ingesting sucrose or isomaltulose drink after standard lunch, followed by satiety assessment	Isomaltulose (50 g)	Sucrose (50 g)	[28]
SB Randomized CS	Singapore	Healthy	Ingesting different sucrose: isomaltulose ratios over 9 test sessions	Various sucrose:isomaltulose ratios	Standard glucose solution	[29]
SB Randomized CS	Singapore	Healthy	Ingesting sucrose or isomaltulose drink (50 g carbohydrate)	isomaltulose (50 g)	Sucrose (50 g)	[11]
DB Randomized CS	Germany	T2DM	Ingesting sugars dissolved in 200 mL water within 5 min after an overnight fast	Isomaltulose	Sucrose	[24]
RCS	Germany	T2DM	Digital nutrition program with 14-day continuous glucose monitoring	Isomaltulose-sweetened meals	Sucrose-sweetened meals	[19]
DB Randomized CS	Thailand	T2DM	Breakfast meal with cooked rice, fried boiled egg with tamarind sauce, Chinese cabbage plus minced pork soup, hot cocoa drink	51% Isomaltulose	Sucrose	[23]
Randomized CS	Denmark	T1DM	Ingesting isomaltulose before or during exercise, glucose and insulin levels measured	Isomaltulose (0.75 g/kg BMI)	Sucrose	[30]

Study design, population, intervention methods, and control conditions for trials investigating isomaltulose versus sucrose. Randomized controlled trials (RCTs) and crossover studies were conducted across various regions and populations. Intervention protocols varied, including direct ingestion of sugars or dietary assessments. The *p*-value for the study is 0.001. (DB: Double-blinded, SB: Single-blinded, RCS: Retrospective Crossover study, CS: Crossover Study.

**Table 3:** Forest plot for plasma glucose levels at 60 min post-meal.

	Group 1: Isomaltulose			Group 1: Sucrose (Control)			Mean Difference	
Study of Subgroup	Mean (mg/dL)	SD (mg/dL)	Total	Mean (mg/dL)	SD (mg/dL)	Total	Weight	IV, Fixed, 95% CI (mg/dL)
Ang et al. [24]	104.4	1.8	11	117	1.8	11	15.70%	−12.6 (−14.10, −11.10)
Lee [29]	24.5	3.9	65	30.7	4.5	65	17.00%	−6.20 (−7.65, −4.75)
Kawai et al., 1985 [26]	110.9	4.9	8	143.3	8.8	8	0.70%	−32.40 (−39.38, −25.42)
Kawai et al., 1989 [8]	195	11	10	237	12	10	0.30%	−42.00 (−52.09, −31.91)
Komindr et al. [23]	87.18	30.78	11	106.18	26.24	11	0.10%	−19.00 (−42.90, 4.90)
Kordowski et al. [19]	28.4	3.6	117	35.2	4.5	117	32.60%	−6.80 (−7.84, −5.76)
Tan [11]	22.9	3.2	50	30.1	4.8	50	13.90%	−7.20 (−8.80, −5.60)
McCarthy et al. [30]	27.5	3.8	8	34.1	4.9	8	1.90%	−6.60 (−10.90, −2.30)
Mills et al. [28]	24	4.2	77	30.8	5.6	77	14.50%	−6.80 (−8.36, −5.24)
Amano et al. [27]	122.5	3.5	10	29.6	4.1	10	3.20%	−7.10 (−10.44, −3.76)
Total			367			367	100%	−7.99 (−8.58, −7.39)

Note: Test for overall effect: Z = 26.26 (*p* < 0.00001); Test for subgroup differences: Not applicable; Heterogeneity: Chi^2^ = 142.23, *df* = 9 (*p* < 0.00001); *I*^2^ = 94%.

## Data Availability

All data supporting the findings of this study are available within the article. The datasets generated or analyzed, including extracted summary statistics from included studies, are available upon reasonable request from the corresponding author. No new datasets were generated or collected from human participants for this study.
